# Domain adaptation for EEG-based, cross-subject epileptic seizure prediction

**DOI:** 10.3389/fninf.2024.1303380

**Published:** 2024-02-02

**Authors:** Imene Jemal, Lina Abou-Abbas, Khadidja Henni, Amar Mitiche, Neila Mezghani

**Affiliations:** ^1^Centre EMT, Institut National de la Recherche Scientifique, Montréal, QC, Canada; ^2^Institute of Applied Artificial Intelligence (I2A), Université TÉLUQ, Montréal, QC, Canada; ^3^Laboratoire de Recherche en Imagerie et Orthopédie, Centre de recherche du CHUM, Montréal, QC, Canada

**Keywords:** epileptic seizure prediction, deep learning, domain adaptation, EEG, cross-subject modeling

## Abstract

The ability to predict the occurrence of an epileptic seizure is a safeguard against patient injury and health complications. However, a major challenge in seizure prediction arises from the significant variability observed in patient data. Common patient-specific approaches, which apply to each patient independently, often perform poorly for other patients due to the data variability. The aim of this study is to propose deep learning models which can handle this variability and generalize across various patients. This study addresses this challenge by introducing a novel cross-subject and multi-subject prediction models. Multiple-subject modeling broadens the scope of patient-specific modeling to account for the data from a dedicated ensemble of patients, thereby providing some useful, though relatively modest, level of generalization. The basic neural network architecture of this model is then adapted to cross-subject prediction, thereby providing a broader, more realistic, context of application. For accrued performance, and generalization ability, cross-subject modeling is enhanced by domain adaptation. Experimental evaluation using the publicly available CHB-MIT and SIENA data datasets shows that our multiple-subject model achieved better performance compared to existing works. However, the cross-subject faces challenges when applied to different patients. Finally, through investigating three domain adaptation methods, the model accuracy has been notably improved by 10.30% and 7.4% for the CHB-MIT and SIENA datasets, respectively.

## 1 Introduction

Epilepsy is a neurological disorder which causes recurrent seizures resulting from brain dysfunction. Symptoms of seizures vary greatly among patients and can range from brief disruptions in activity to loss of consciousness and severe convulsions. To diagnose epilepsy, physicians use electroencephalography (EEG), which records the electrical activity of the brain using electrodes placed on the skull. Studies have shown that there is a pre-ictal period, lasting several minutes before the onset of a seizure. During the pre-ictal EEG recordings display patterns that are different from those of the seizures and also from normal periods, called inter-ictal (Mormann et al., 2005). Thus, by distinguishing pre-ictal from inter-ictal states, it is possible to predict seizures.

Computer-aided models for seizure prediction can be grouped into three categories: (1) patient-specific modeling, which is tailored to each individual patient, (2) multiple-subject modeling, also called patient-independent modeling, which is applied to a dedicated set of patients, and (3) cross-patient modeling, a generalized model that uses data from multiple patients and can be applied to new, unseen patients.

1. **Patient-specific modeling** involves using a portion of a single patient's data for model training and the remaining data for performance evaluation. However, this type of model is not practical as it is limited by the amount of data available and the need to record a sufficient number of seizures for each individual patient.
2. **Multiple-subject modeling**, also called patient-independent modeling, is a complex task that predicts seizures for subjects in a dedicated ensemble of subjects. This modeling does not have the limitation of lack of data as it utilizes all patient data grouped into at least two sets to train and test the model. However, this approach faces the major challenge of adapting the prediction model to new data from unseen patients.
3. **Cross-subject modeling**, also known as generalized modeling, is the most complex type for seizure prediction. This approach allows generalization to other patients and does not require labeled data for new patients.

Research has mostly focused on multiple-subject modeling for seizure prediction (Khan et al., 2017; Tsiouris et al., 2017; Dissanayake et al., 2021a). As far as we know, cross-subject seizure prediction has not been investigated, although cross-subject modeling has been successfully applied to other tasks, such as seizure detection (Zhang et al., 2020), emotion recognition (Li et al., 2018), and mental load assessment (Albuquerque et al., 2019). Cross-subject modeling of seizure prediction is justified by the high variability between the data of different patients (Jemal et al., 2022). Indeed, data from a new patient may differ significantly from data of patients whose data served to train the cross-subject model. This is often referred to as a domain shift (Ben-David et al., 2010). It is common in real data applications, and can result in a significant drop in classification performance (Ponce et al., 2006). To address this issue, domain adaptation can be used. In this context, a domain refers theoretically to the probability distribution from which the problem data are drawn. The training dataset is called the source domain data and the test dataset is called the target domain data. Domain adaptation uses labeled source data and unlabeled target data to learn a model that performs well in both the target and source domains. This is generally achieved by re-weighting the source samples to minimize the distribution shift, so that the source samples closest to the target domain are given more importance (Huang et al., 2006; Sugiyama et al., 2007). Alternatively, one can use a pre-trained model from the source domain on the new target domain (Oquab et al., 2014). This approach, known as a parameter-based approach, can only be applied if some labeled data are available in the target domain. Another approach on which we focus in this study, called feature-based (Daumé III, 2009; Ganin et al., 2016; Long et al., 2018) uses the source and target data to learn features that display similar behavior in classification on both the source and the target domains data. The use of domain adaptation to classify EEG data has been successful in applications such as emotion recognition (Li et al., 2019; Ma et al., 2019; Zhang W. et al., 2019), motor imagery classification (Wu et al., 2019; Tang and Zhang, 2020), and evaluation of sleep quality (Zhang et al., 2017).

In this paper, we studied epileptic seizure prediction. We began by developing and investigating a new method of multiple-subject prediction by deep learning, and compared it to the state-of-the-art of such methods. Multiple-subject modeling broadens the scope of patient-specific modeling to account for the data from a fixed set of patients, thereby providing some useful level of generalization. To enhance seizure prediction and increase generalization across data from new patients, we have developed and investigated cross-subject model. We explored both model with and without domain adaptation, allowing us to assess the impact of data variability and underscore the significance of domain adaptation. Results show a significant improvement in accuracy, F1-score, and Area under the curve, on both CHB-MIT and SIENA datasets.

The remainder of this paper is organized as follows: Section 2 provides a summary of previous research on seizure prediction. Section 3 describes the EEG databases, the deep neural network architecture, as well as the domain adaptation methods used. Section 4 presents the experimental setup and results. Section 5 contains a conclusion and alludes to future directions of research.

## 2 Related work

Existing seizure prediction models fall into two major categories: the general multiple-subject modeling that applies to all patients, and the patient-specific modeling that addresses each patient individually. There has been little recent work on multiple-subject prediction modeling. The study (Tsiouris et al., 2017) compared different classification algorithms for seizure prediction, including the repeated incremental pruning to produce error reduction (RIPPER) algorithm, support vector machines (SVM), and neural networks (NN). The main objective was to distinguish between pre-ictal and inter-ictal EEG segments in data from multiple patients. Using a balanced number of selected pre-ictal and inter-ictal records from each patient in the CHB-MIT database, the SVM was found to have the best results with an accuracy of 68.5%. More recent studies, such as Khan et al. (2017), have demonstrated improved results using a convolutional neural network (CNN) on the wavelet transform of EEG signals, to achieve a sensitivity of 87.8% with a low false prediction rate of 0.142 FP/h. In Dissanayake et al. (2021a), a multi-task deep learning approach was used for both seizure classification and patient prediction using a Siamese network architecture. Using the CHB-MIT-EEG dataset, they reported an average accuracy of 91.54%. Another study, (Wu et al., 2022), utilized knowledge distillation to transfer information from a multiple-subject model trained on data from *N*−1 patients to a patient-specific model trained on the remaining patient's data. This approach led to improved patient-specific prediction results compared to four other existing methods, with an average improvement of 3.37% in accuracy, 2.33% in sensitivity, and a reduction in false predictions by an average of 0.044/h when tested on 11 patients from the CHB-MIT dataset.

Recently, deep learning has gained attention for its application in seizure prediction. For instance, a study by Tsiouris et al. (2018) employed a deep Long Short-Term Memory (LSTM) network to predict seizures using EEG segments with a pre-ictal duration of 120 minutes. The study reported high sensitivity and specificity of 99.84% and 99.86%, respectively, using the CHB-MIT dataset. Other studies have applied CNNs, such as (Truong et al., 2018) which used spectrogram representations of EEG data, and Zhang Y. et al. (2019) which applied a common spatial algorithm model prior to the CNN. Another recent study (Zhao et al., 2020) proposed a one-dimensional CNN trained on raw EEG data trained with raw EEG data to predict seizure occurrence. The study reported an area under the curve, sensitivity, and false prediction rate of 0.915, 89.26%, 0.117/h and 0.970, 94.69%, 0.095/h on american epilepsy society (AES) and CHB-MIT data, respectively. An alternative study proposed adversarial training for data augmentation to account for the limited amount of pre-ictal data, which improved the performance and robustness of the model. Recently, there has been a focus on developing interpretable models for patient-specific seizure prediction, as seen in studies such as Jemal et al. (2022) and Pinto et al. (2021), which utilize a genetic algorithm and a deep learning classifier, respectively.

## 3 Materials and methods

### 3.1 Datasets and pre-processing

In this study, experiments were conducted using two open-access datasets, the SIENA EEG database and the CHB-MIT dataset. The datasets description is summarized in Table 1.

**Table 1:** Overview of SIENA and CHB-MIT datasets used in this study for seizure prediction.

	**SIENA**	**CHB-MIT**
Number of subjects	14	23
Age of subjects	20–71	1.5–19
Number of seizures	47	198
Type of recordings	Scalp	Scalp
Total hours of EEG recordings (h)	128	940
Number of channel	29	23
Sampling frequency (Hz)	512	256

The SIENA dataset (Detti et al., 2020), acquired at the unit of neurology and neurophysiology of the university of SIENA, contains recordings from 14 epileptic subjects aged 20 to 71 years. The subjects were monitored using video EEG. A total of 29 EEG channels sampled at 512 Hz were recorded following the standard 10–20 system. During 128 hours of EEG recording, 47 epileptic seizures were recorded. The time of the onset of a seizure and its duration were identified by experts. The CHB-MIT dataset (Shoeb, 2009), collected at Boston children's hospital, contains 940 h of long-term continuous multichannel scalp EEG recordings from 23 epileptic subjects aged 1.5 to 19 years. A minimum of 19 EEG channels sampled at 256 Hz were recorded according to the international 10/20 standard. In total, these recordings included 198 seizures in which the onset and the end were precisely annotated by clinicians with expertise in neuroscience. In this study, we eliminated recordings with fewer than 23 electrodes.

The raw EEG channels from both datasets were filtered to focus on frequencies relevant to epilepsy analysis and to eliminate noise sources. This was done by using a notch filter with a cutoff frequency of 50Hz and a band-pass filter with a bandwidth of 0.5–70Hz. The pre-ictal period, which is the time before a seizure starts, was set to 1 hour based on published literature. Moreover, the post-ictal period, which is the time after the seizure ends, was eliminated to exclude any effects (Daoud and Bayoumi, 2019; Dissanayake et al., 2021a). Non-overlapping windows of 10 s were extracted from inter-ictal and pre-ictal recordings. To address the limited number of pre-ictal samples, under-sampling was used to randomly select examples from the majority class. The windows were then normalized so that the channels had zero mean and unit standard deviation. This resulted in a total of 77,529 inter-ictal samples and 89,783 pre-ictal samples from the CHB-MIT dataset and 197,805 inter-ictal samples and 80,845 pre-ictal samples from the SIENA dataset, which were split between training, validation, and testing data.

### 3.2 Deep learning architecture

**Figure 1:**
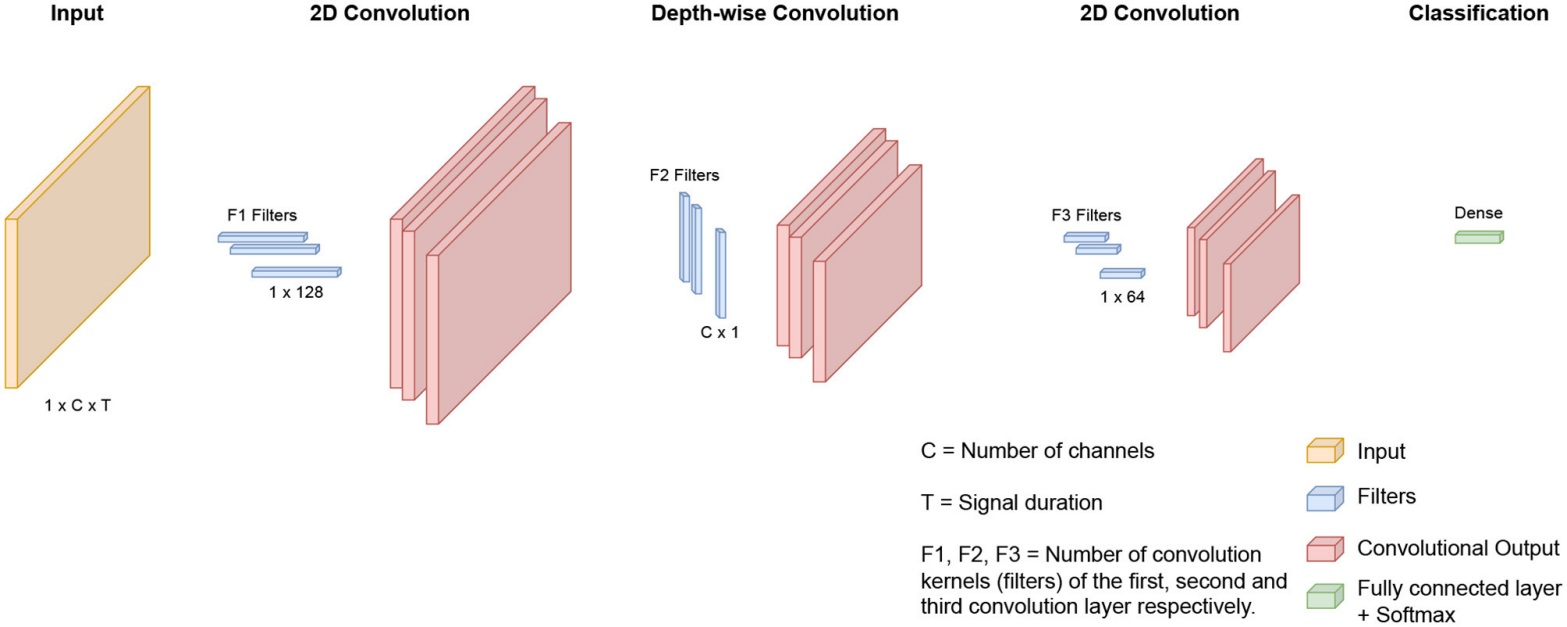
Illustration of the deep-learning architecture: The network processes EEG inputs with a standard 2D convolution to learn frequency filters. Next, depth-wise convolution is applied to learn spatial filters. Finally, features are extracted with a 2D convolution, and the outputs proceed through a fully connected layer with Softmax activation.

The architecture used for the multiple-subject and cross-subject models in this study was previously proposed by our team for the prediction of patient-specific seizures, as outlined in Jemal et al. (2022). We used the same network architecture. However, in contrast to the work in Jemal et al. (2022), which focuses on patient-specific modeling, our study centers around both multiple-subject modeling and cross-subject modeling. Additionally, we integrated domain adaptation techniques to enhance overall performance. As shown in Figure 1 and Table 2, the network consists of a three-layer convolutional neural network designed to be interpretable. The first layer uses standard 2D convolutions to extract relevant frequency components of the signal. The second layer uses depth-wise filters, which is the application of convolution filters to each feature map (output from the previous layer) independently from the other maps. This step allows learn spatial filters from the previous outputs. The first and second steps are similar to the Filter Bank Common Spatial Pattern (FBCSP) algorithm commonly used for EEG data encoding. The third 2D convolutional layer is used for feature extraction. Finally, the output is passed through a fully connected layer with a Softmax activation function.

**Table 2:** The detailed architecture of the network, where C = number of channels, T = signal duration, F1 = number of convolution kernels filters to learn frequency filters, F2 = number of convolution kernels to learn spatial filters, F3 = number of convolution kernels for feature extraction, N = number of classes, respectively.

**Layer**	**#Filter**	**Filter size**	**#Parameters**	**Output**	**Activation**
Input				(1,C,T)	
2D Convolution	F1	(1,128)	128 * F1	(F1,C,T)	Linear
Batch normalization	2*F1			(F1,C,T)	
Depth-wise convolution	F2*F1	(C,1)	C*F2*F1	(F2*F1,1,T)	
Batch normalization			2*F2*F1	(F2*F1,1,T)	
Activation				(F2*F1,1,T)	Relu
Average-pooling		(1,16)		(F2*F1,1,T//16)	
Dropout				(F2*F1,1,T//16)	
2D Convolution	F3	(1,64)	64*F3	(F3,F2*F1,T')	Linear
Batch normalization			2*F3	(F3,F2*F1,T')	
Activation				(F3,F2*F1,T')	Relu
Average-pooling		(1,16)		(F3,F2*F1,T'//16)	
Dropout				(F3,F2*F1,T'//16)	
Linear (flatten)				(F3*F2*F1*(T'//16))	
Dense				N = 2	Softmax

### 3.3 Multiple-subject vs. cross-patient modeling

**Figure 2:**
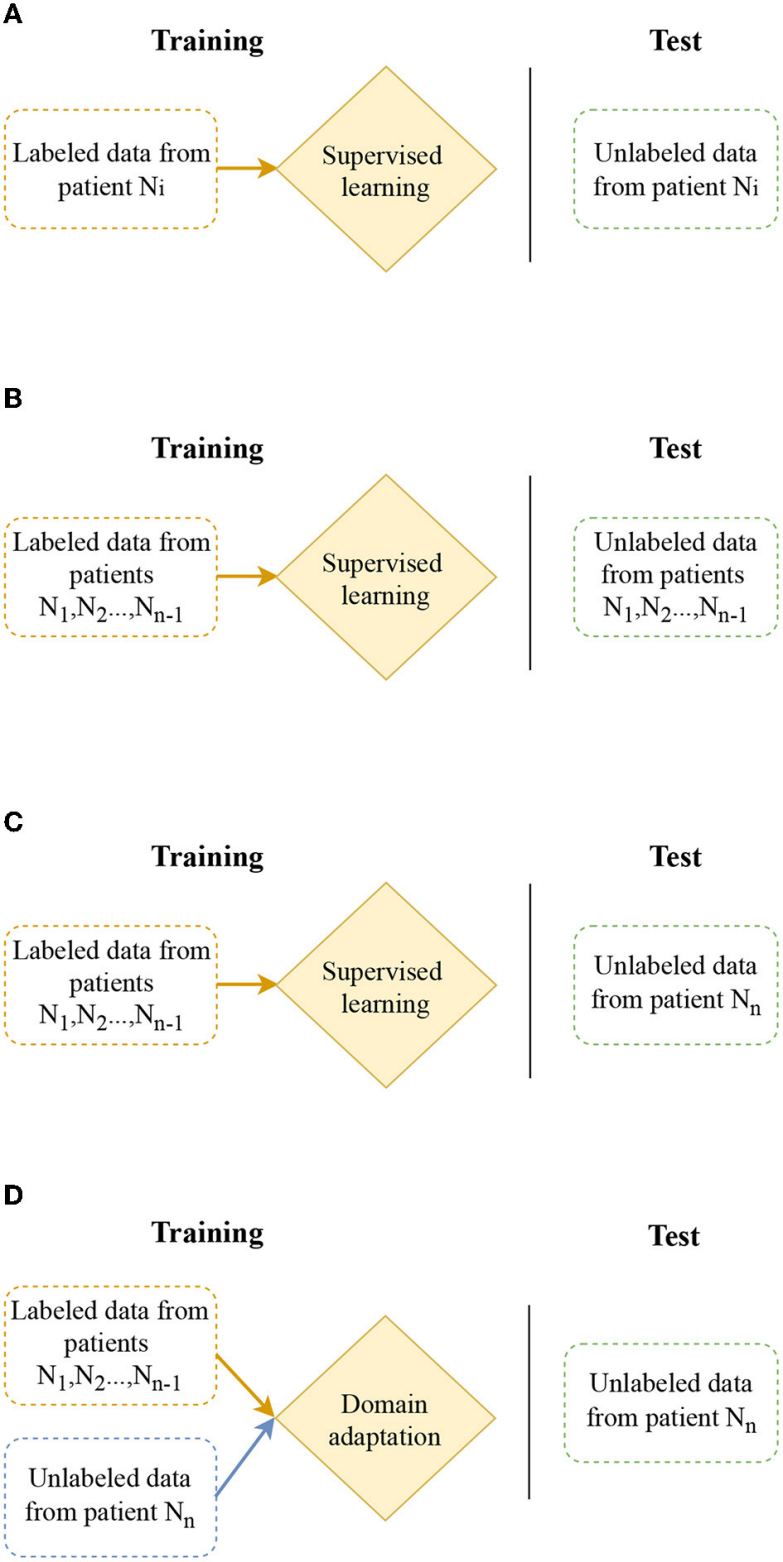
Different modeling for seizure prediction: patient-specific modeling, multiple-subject modeling, cross-patient modeling, and cross-patient modeling with domain adaptation. **(A)** Patient-specific modeling: design model which focus on individual patient. **(B)** Multiple-subject modeling: design model considering data from different patients. **(C)** Cross-patient modeling: design model which generalize to a new patient. **(D)** Cross-patient modeling with domain adaptation: improve model to work effectively across different patients through domain adaptation.

The task of seizure prediction can be approached using several models as depicted in Figure 2. Patient-specific modeling involves using data from a single patient to train a unique model for that patient, as shown in Figure 2A. A more practical solution is multiple-subject modeling (Figure 2B), which uses data from multiple patients grouped into training and test sets to learn a single model that can be applied to all patients. However, this model may not generalize well to new patients. The focus of this work is cross-subject modeling (Figure 2C), which involves using labeled data from *N*−1 patients for training and data from the remaining patient for testing.

This study investigates domain adaptation methods to improve cross-subject modeling (Figure 2C). Domain adaptation involves using labeled data from *N*−1 patients (source domain) and unlabeled data from a new patient (target domain) to transfer the model. With this method, the model is able to perform well on both the *N*−1 patients used for training and the new patient. To accomplish this, we adopt a feature-based approach, which aims at learning features allowing good classification in both the source domain and the target domain. We investigate our architecture with three different domain adaptation algorithms: Discriminative Adversarial Neural Network (DANN), Domain Adversarial Conditional Adaptation (CDAN), and the Entropic conditioning variant of CDAN (CDAN+E). The selection of Domain-Adversarial Neural Network (DANN), Conditional Domain Adversarial Network (CDAN), and Conditional Domain Adversarial Network with Entropy minimization (CDAN+E) is motivated by their effectiveness in addressing domain shift challenges in previous studies (Du et al., 2020; Tang and Zhang, 2020; Li et al., 2022). DANN is chosen for its capacity to learn domain-invariant features, while CDAN incorporates conditional information for complex relationships between source and target domains. CDAN+E further enhances CDAN by integrating entropy minimization to boost model confidence in predictions.

### 3.4 Domain adaptation and cross-subject generalization

#### 3.4.1 Supervised learning

Let *X* be the input space, the set of all possible examples or data points and *Y* be its corresponding label space. For example, *Y* would be {0, 1} for a binary classification case. A domain *D* is defined as a distribution over *X*.

Moreover, let *F*:*X*→*Y* be a deterministic mapping function such as a neural network. In general, the quality of the predictor *F*(*x*) can be measured using loss function *l*(*F*(*x*), *y*). Supervised learning (Duda et al., 1973) can be defined as searching the optimal predictor F^*^ using the optimization problem of the following form


(1) $$\begin{array}{l} \underset{F}{\min}L_{task}\left(F\left(X\right),y\right) \end{array}$$

The training data are used to find the optimal predictor F^*^, and the test data for the evaluation. Generally, the training and test data are assumed drawn from the same distribution. However, this assumption often does not hold in practice, thus justifying domain adaptation.

#### 3.4.2 Domain adaptation

Let *X*_*S*_ be the source domain data (training data) drawn from the distribution *P*_*S*_(*X*_*S*_) and the target domain data (test data) denoted as *X*_*T*_ are drawn from the distribution *P*_*T*_(*X*_*T*_).

In the training stage, we assume in the training stage of sufficient labeled source domain data $D_{S}=\left\{\left(x_{i}^{S},y_{i}^{S}\right)\right\}$ and unlabeled target domain data, $D_{T}=\left\{\left(x_{i}^{T},y_{i}^{T}\right)\right\}$ . The input spaces and label spaces between domains are assumed the same: if *x*_*S*_ = *x*_*T*_, then *y*_*S*_ = *y*_*T*_. However, due to the data shift, *P*_*S*_(*X*_*S*_)≠*P*_*T*_(*X*_*T*_) and *P*_*S*_(*Y*_*S*_/*X*_*S*_)≠*P*_*t*_(*Y*_*S*_/*X*_*T*_).

The objective of domain adaptation is to adjust a model trained on a source domain to perform effectively on a new target domain. The feature-based approach aims to learn features that minimize the difference between the source and target distributions (Ganin and Lempitsky, 2015). Mathematically, this is expressed as:


(2) $$\begin{array}{l} \underset{\theta ,\phi }{\min}L_{task}\left(D_{S},\theta ,\phi \right)+\lambda L_{domain}\left(D_{S},D_{T},\theta ,\phi \right) \end{array}$$

where $L_{task}$ represents the task-specific loss on the labeled source domain data, $L_{domain}$ is a domain discrepancy measure, and λ is a balancing parameter.

We will discuss three different methods for achieving this: the Discriminative Adversarial Neural Network (DANN), the Domain Adversarial Conditional Adaptation (CDAN), and the Entropic conditioning variant of CDAN (CDAN+E).

#### 3.4.3 Discriminative adversarial neural network

**Figure 3:**
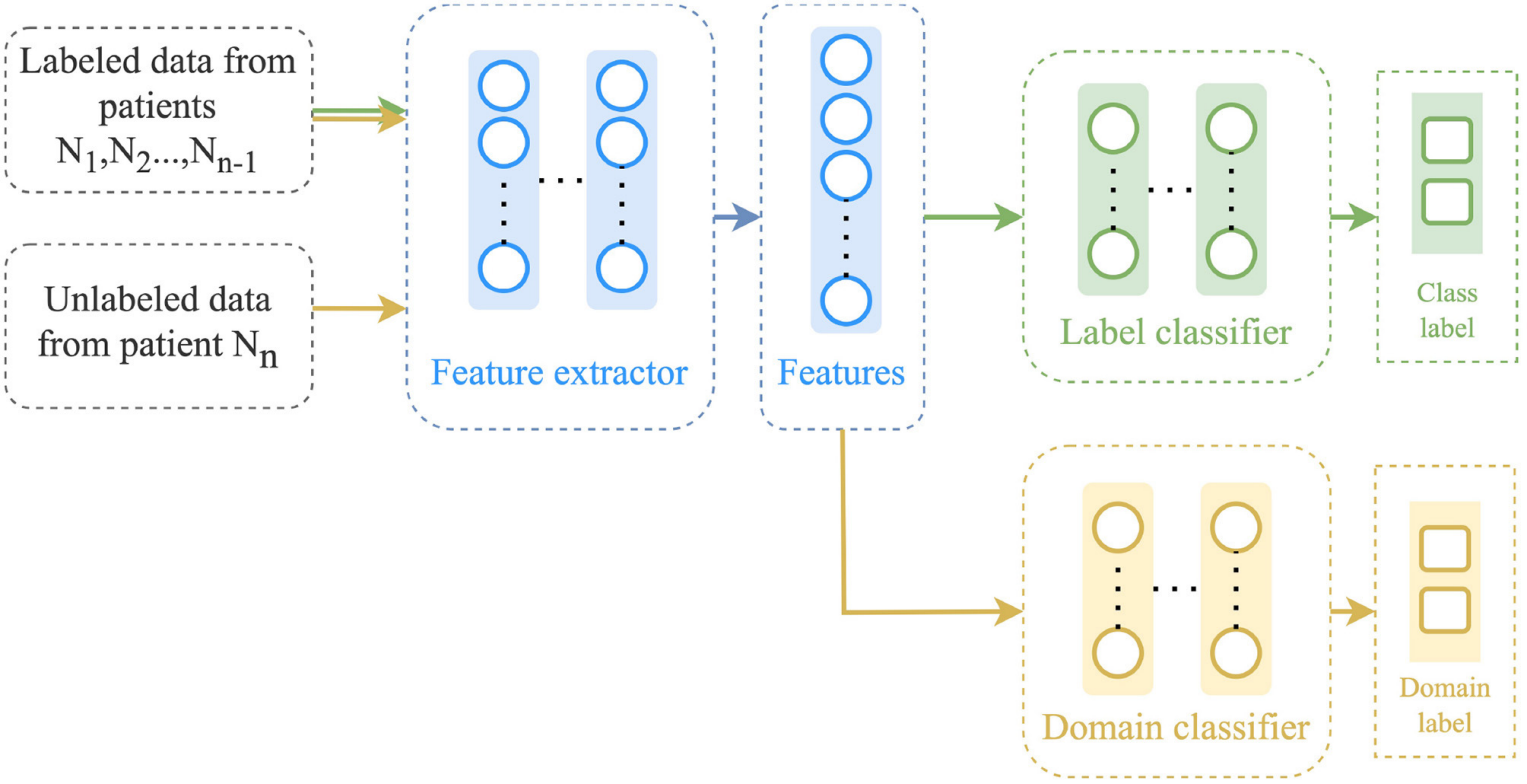
Architecture of the discriminative adversarial neural network (DANN).

The DANN method, as described by Ganin et al. (2016), aims at learning a feature representation that is discriminative for the classification task on the source domain and not so regarding the shift between domains. It is based on the assumption that unlabeled target domain data is available. As shown in Figure 3, it consists of three main components: a feature extractor ϕ, a label predictor *F* and a domain discriminator *D*. The feature extractor ϕ also referred to as the generator, is a neural network that is trained using data from both the source and target domains to learn a feature representation that is not specific to any particular domain. The label predictor *F* is trained to minimize the classification error on the source domain data, while the domain discriminator *D* is trained to differentiate between the source domain and the target domain. The label predictor and the domain discriminator work adversarially, encouraging the feature extractor to learn domain-invariant representations. The parameters of all three components are optimized according to the following objective function:


(3) $$\begin{array}{l} \begin{array}{c} \underset{\phi ,F}{\min}L_{task}\left(F\left(\phi \left(X_{S}\right)\right),y_{S}\right)-\lambda \left(log\left(1-D\left(\phi \left(X_{S}\right)\right)\right)+log\left(D\left(\phi \left(X_{T}\right)\right)\right)\right)\\ \underset{D}{\max}log\left(1-D\left(\phi \left(X_{S}\right)\right)\right)+log\left(D\left(\phi \left(X_{T}\right)\right)\right), \end{array} \end{array}$$

where λ is a trade-off parameter.

#### 3.4.4 Conditional domain adaptation network and Entropic conditioning variant of CDAN (CDAN+E)

**Figure 4:**
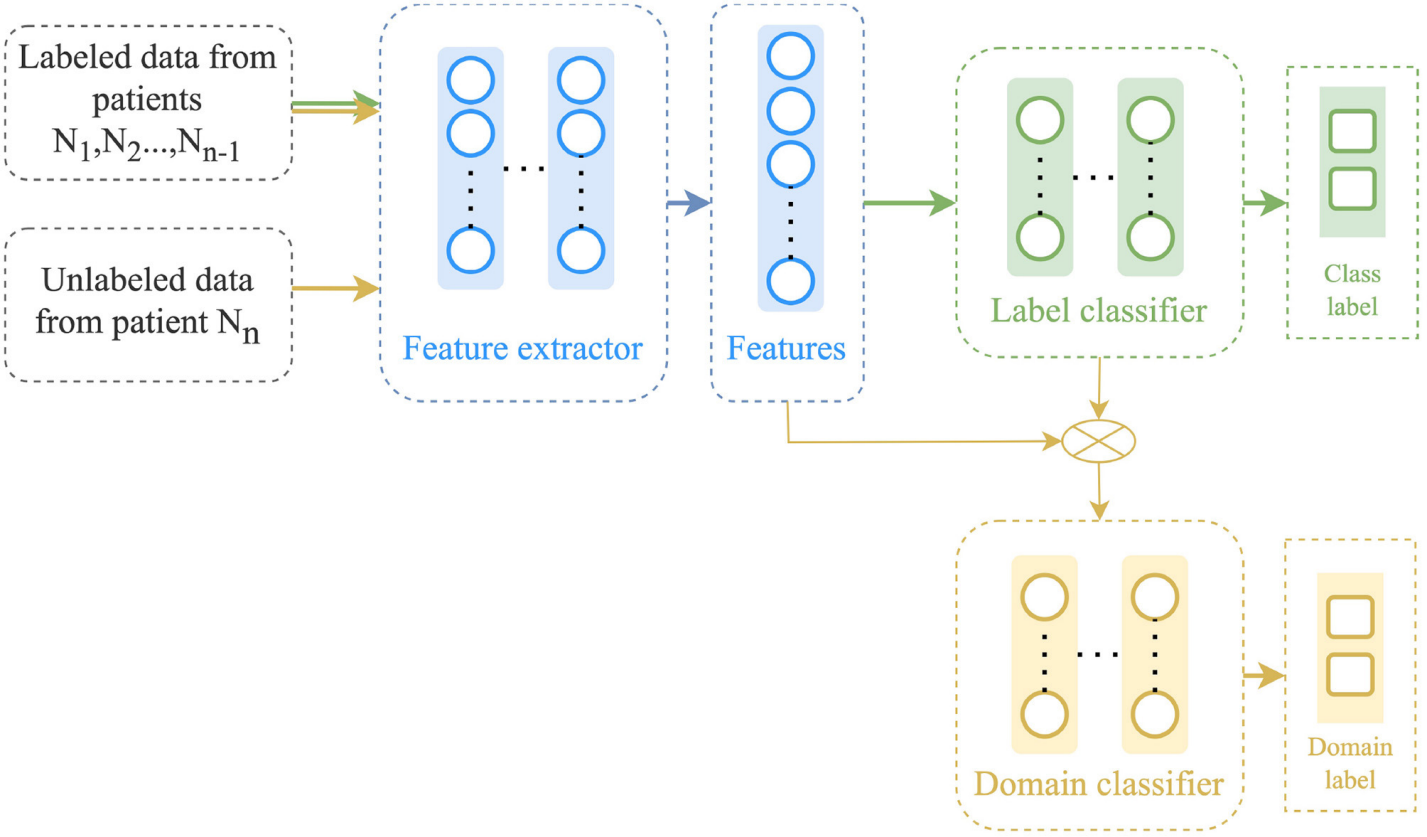
Architecture of the conditional domain adaptation network (CDAN).

The CDAN approach, proposed by Long et al. (2018), is similar to the DANN approach and also contains three main components: a feature extractor ϕ, a label predictor *F*, and a domain discriminator *D* as illustrated in Figure 4. However, in CDAN, a conditional discriminator *D* is used through the joint variable *H* = (ϕ, *F*) in order to improve discriminability by capturing the cross-covariance between feature representations and classifier predictions. The method uses a multilinear conditioning strategy to combine the feature vector with the predicted label. The label predictor and domain discriminator are trained alternatively to minimize the label classification and domain classification losses, respectively. The optimization formulation for CDAN is as follows:


(4) $$\begin{array}{l} \min_{\phi ,F}L_{task}(F(\phi (X_{S})),y_{S})-\lambda (log(1-D(\phi (X_{S})⊗F(\phi (X_{S}))+\\ log(D(\phi (X_{T})⊗F(X_{T})))\\ \max_{D}log(1-D(\phi (X_{S})⊗F(X_{S}))+log(D(\phi (X_{T})⊗F(X_{T})) \end{array}$$

where λ is a trade-off parameter, and, ϕ(*X*_*S*_)⊗*F*(*X*_*S*_) is the multilinear map between the encoded sources and the task predictions.

In addition, an extension of the CDAN algorithm, known as CDAN+E, was also proposed by Long et al. (2018), in which an entropy conditioning strategy was introduced to improve transferability. This approach involves using a score that quantifies the uncertainty of the classifier predictions. The score is based on an entropy criterion, and it is used to re-weight each example used by the conditional domain discriminator. This helps obtain better transferability.

## 4 Results

Performance evaluation of this study multiple-subject and cross-patient models for EEG based seizure prediction was conducted on CHB-MIT and SIENA datasets. Pytorch (Paszke et al., 2017) was used to implement the proposed architecture. Data pre-processing was done using the MNE-Python package (Gramfort et al., 2013). Across all models, we employed the gradient-based ADAM optimizer with coefficients β_1_ and β_2_ set to 0.9 and 0.999 respectively for its efficiency and reliability in reaching a global minimum. The learning rate was set to 0.005. To prevent over-fitting, we used a holdout validation method to divide the data into a validation set and a training set, and the training. The training stopped after 500 epochs or when the validation loss remained constant for at least 20 epochs. To evaluate the models, we used various metrics including accuracy, precision, recall, F1-score, as well as the receiver operating characteristic (ROC) and the area under the curve (AUC).

### 4.1 Multiple-subject modeling

**Figure 5:**
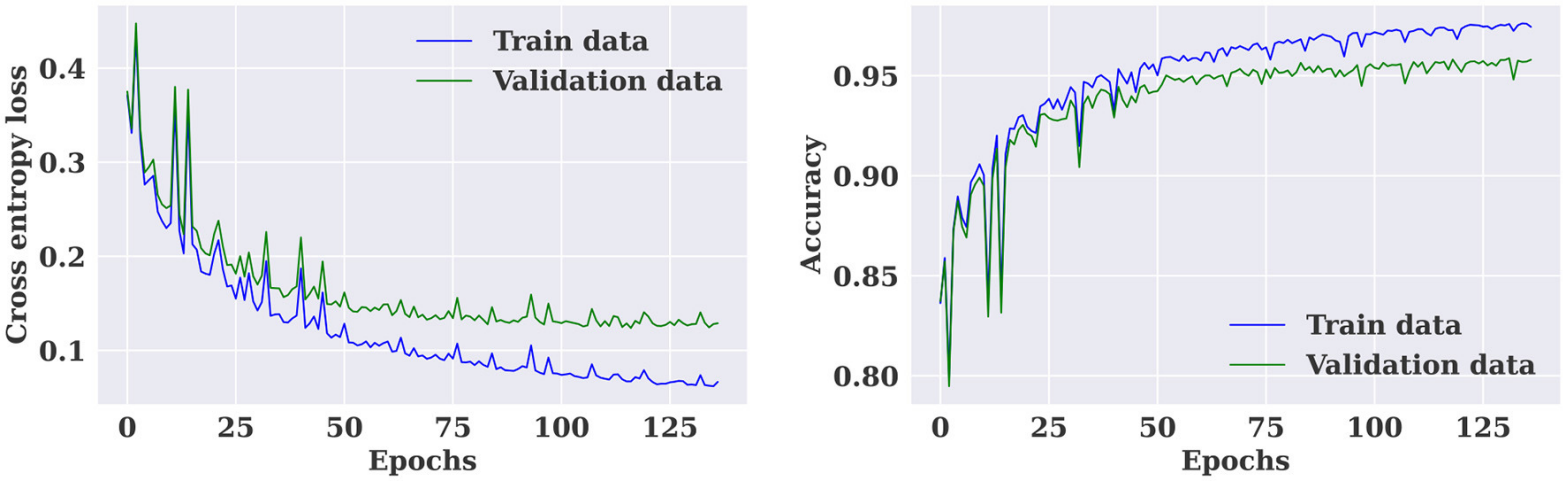
Training and validation loss and accuracy curves for the multiple-subject seizure prediction model trained using SIENA dataset.

**Figure 6:**
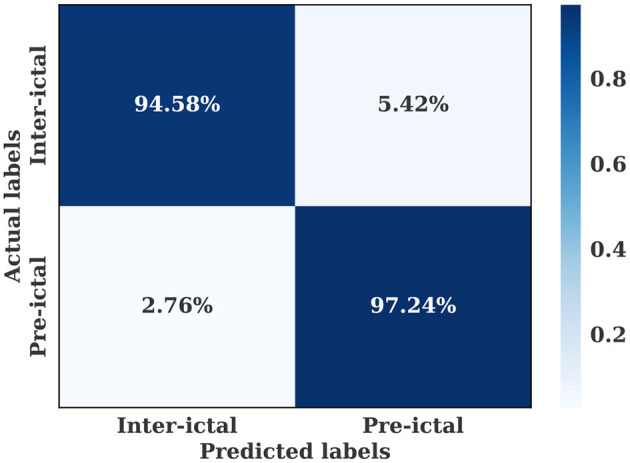
Confusion matrix for the multiple-subject seizure prediction model trained using SIENA dataset.

The multiple-subject model was evaluated using data from all patients in the SIENA dataset, which was divided into training, validation, and test sets. The model achieved a high accuracy of 96.01%, sensitivity of 97.24%, and specificity of 94.57%. The model also produced a high AUC value of 0.96. The training and validation loss curves indicate that the model does not suffer from over-fitting, as shown in Figure 5. The confusion matrix in Figure 6 demonstrates the model's classification performance. Comparison with current state-of-the-art seizure prediction models, as shown in Table 3, indicates that the model has comparable performance.

**Table 3:** Comparisons of state-of-the-art seizure prediction methods applied on SIENA dataset using a multiple-subject modeling.

**References**	**Year**	**Method**	**Accuracy (%)**	**Sensitivity (%)**	**Specificity (%)**
Dissanayake et al. (2021b)	2022	Geometric DL	95.56	95.33	95.11
Dissanayake et al. (2021b)	2022	Geometric DL	96.05	96.05	96.61
This work	2022	CNN	96.01	97.24	94.57

**Figure 7:**
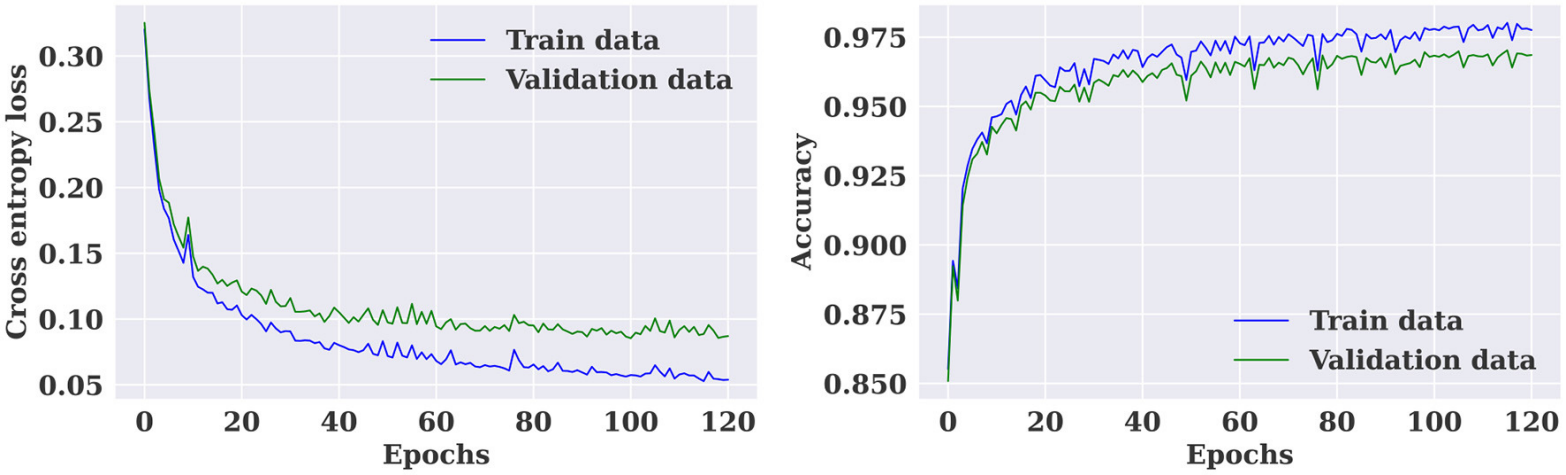
Training and validation loss and accuracy curves for the multiple-subject seizure prediction model trained using CHB-MIT dataset.

**Figure 8:**
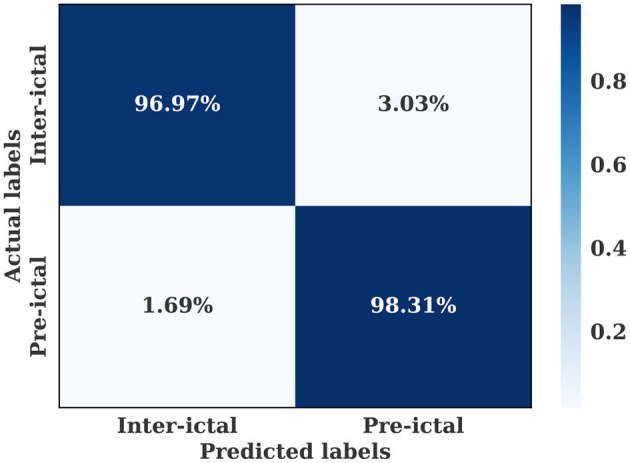
Confusion matrix for the multiple-subject seizure prediction model trained using the CHB-MIT dataset.

The multiple-subject model showed also high performance when evaluated using the CHB-MIT dataset, with an accuracy of 97.36%, a sensitivity of 98.31%, and a specificity of 96.97%. As shown in Figure 7, the training and validation loss decreased to a stable point, with a small gap between the training and validation curves, indicating that the model is well-fitting. The confusion level is relatively low, as indicated by the percentages of confusion between inter-ictal and pre-ictal instances (3.03% and 1.69%, respectively) as shown in Figure 8. According to Table 4, the model outperforms current state-of-the-art models evaluated on the same dataset.

**Table 4:** Comparison of the state-of-the-art seizure prediction methods applied on the CHB-MIT dataset using a multiple-subject modeling.

**References**	**Year**	**Method**	**Accuracy (%)**	**Sensitivity (%)**	**Specificity (%)**
Tsiouris et al. (2017)	2017	SVM	68.50	81.20	-
Khan et al. (2017)	2017	CNN	-	87.80	-
Dissanayake et al. (2021a)	2021	Multitask DL	91.50	92.45	89.94
Dissanayake et al. (2021b)	2022	Geometric DL	95.38	94.47	94.26
Dissanayake et al. (2021b)	2022	Geometric DL	95.02	95.94	93.52
This work	2022	CNN	97.36	98.31	96.97

### 4.2 Cross-subject modeling

In contrast to the multiple-subject modeling approach, the cross-subject modeling employs a validation strategy called leave-one-patient-out. This strategy involves using data from each patient in the dataset, one at a time, for testing while training the classifier with data from the remaining *N*−1 patients. This method is commonly used to assess the ability of the classifier to generalize to new patients.

We evaluated the proposed cross-subject method using both data sets by measuring performance in terms of F1-score, accuracy, and AUC. The results, presented in Tables 5, 6, show the evaluation results for each patient in the SIENA and CHB-MIT datasets respectively. The overall averages across all patients in the SIENA dataset were 39.81% for F1-score, 48.69% for accuracy, and 0.48 for AUC. Results were slightly better with the CHB-MIT dataset which uses more patients, with averaged F1-score, accuracy, and AUC, equal to, respectively, 55.34%, 63.5%, and 0.69. The performance degradation can be attributed to the mismatch between the distribution of the new patient data and the training distribution. This issue is particularly pronounced in seizure prediction, as most studies in this field focus on patient-specific models. Additionally, our previous research (Jemal et al., 2021) on the complexity of EEG features in predicting epileptic seizures, highlighted the significant variability in EEG data between patients. To mitigate the potential data shift, we explored three domain adaptation methods: DANN, CDAN, and CDAN+E.

**Table 5:** Evaluation of cross-subject modeling using the leave-one-patient out strategy (SIENA dataset).

**Patient id**	**F1-score(%)**	**Accuracy(%)**	**AUC (%)**
PN01	29.75	30.75	0.31
Pn03	53.78	56.27	0.56
PN05	54.76	53.23	0.53
PN06	14.05	40.32	0.34
PN07	28.40	41.04	0.40
PN09	61.39	49.03	0.48
PN11	54.93	64.18	0.67
PN12	30.33	52.49	0.54
PN13	8.58	50.10	0.50
PN14	24.45	50.81	0.51
PN16	56.20	45.67	0.44
PN17	61.14	50.40	0.50
Average	39.81 ± 19.14	48.69 ± 8.53	0.48± 0.09

**Table 6:** Evaluation of cross-subject modeling using leave-one-patient out strategy (CHB-MIT dataset).

**Patient id**	**F1-score(%)**	**Accuracy(%)**	**AUC (%)**
Chb01	15.30	32.93	0.29
Chb02	64.63	52.46	0.55
Chb03	32.74	49.18	0.49
Chb04	40.31	51.78	0.52
Chb05	40.08	59.59	0.67
Chb06	56.73	54.39	0.54
Chb07	67.44	56.58	0.61
Chb08	65.25	52.27	0.55
Chb 09	54.54	55.56	0.55
Chb 10	57.94	54.61	0.55
Chb 11	95.31	95.52	0.96
Chb 13	89.73	90.62	0.92
Chb 14	10.90	52.88	0.76
Chb 15	41.95	63.27	0.79
Chb 16	66.70	74.98	0.83
Chb 17	43.76	63.72	0.78
Chb 18	59.34	71.09	0.82
Chb 19	68.78	76.21	0.84
Chb 20	6.51	51.16	0.63
Chb 21	81.48	84.37	0.88
Chb 22	88.64	89.80	0.91
Chb 23	69.38	64.19	0.66
Average	55.34 ± 24.57	63.5 ± 15.91	0.69 ± 0.17

### 4.3 Domain adaptation for cross-subject seizure prediction

Individual differences in physiological states, neural activity patterns, and EEG signal characteristics among patients can indeed introduce noise and confusion when combining data for model training. To address the problem, we use domain adaptation. Specifically, we use unlabeled data from new patients in learning of domain-invariant features. With domain adaptation, our model learns to discern and adapt to the inherent variations among individual patients, effectively mitigating the impact of noise introduced by inter-subject differences. This approach enables the model to generalize more robustly across patient populations, thereby enhancing its reliability and performance in the presence of accrued physiological and neural characteristics variability. We assessed the effectiveness of three different domain adaptation methods against a baseline cross-subject modeling method using leave-one-patient-out. The results displayed in Tables 7, 8, indicate that all three methods (DANN, CDAN, and CDAN+E) enhanced performance on both SIENA and CHB-MIT datasets. Notably, the CDAN method performed exceptionally well with 60.27% accuracy, 59.77% F1 score, and 0.61 AUC on the SIENA dataset. Significant improvement in performance was also observed on the CHB-MIT dataset with 70.90% accuracy, 66.45% F1-score, and 0.75 AUC for CDAN+E adaptation.

**Table 7:** Domain adaptation for cross-subject modeling: results obtained using DANN, CDAN, and CDAN+E adaptation methods using the leave-one-patient-out strategy on the SIENA dataset.

**PATIENT ID**	**DANN**			**CDAN**			**CDAN+E**		
	**F1 (%)**	**ACC (%)**	**AUC**	**F1 (%)**	**ACC (%)**	**AUC**	**F1 (%)**	**ACC (%)**	**AUC**
PN01	57.18	50.50	0.50	75.48	73.32	0.74	34.56	32.06	0.32
PN03	67.84	57.66	0.62	62.49	65.90	0.66	58.92	59.61	0.60
PN05	54.86	55.64	0.56	57.89	56.91	0.56	59.07	56.86	0.57
PN06	32.586	38.53	0.38	45.67	49.16	0.49	20.02	37.70	0.5
PN07	67.19	51.63	0.66	76.72	73.16	0.75	65.13	57.48	0.59
PN09	66.10	50.54	0.54	65.38	50.11	0.51	65.87	51.62	0.56
PN11	60.94	57.80	0.58	66.17	71.10	0.73	65.87	63.71	0.64
PN12	61.62	55.58	0.56	55.96	56.83	0.57	60.35	60.27	0.60
PN13	24.84	51.56	0.53	11.84	51.46	0.57	11.71	50.88	0.54
PN14	61.56	60.11	0.60	69.67	64.50	0.66	62.73	57.97	0.58
PN16	65.16	48.73	0.39	63.34	52.73	0.54	61.85	45.42	0.32
PN17	60.78	47.86	0.46	66.66	58.10	0.61	56.79	47.86	0.47
Average	56.72 ± 13.74	52.18 ±5.8	0.53 ± 0.08	59.77 ± 17.28	60.27 ± 9.00	0.61 ± 0.09	51.91 ± 18.85	51.79 ± 9.61	0.52 ± 0.10

**Table 8:** Domain adaptation for cross-subject modeling: Results obtained using DANN, CDAN, and CDAN+E adaptation methods using the leave-one-patient-out strategy on the CHB-MIT dataset.

**PATIENT ID**	**DANN**			**CDAN**			**CDAN+E**		
	**F1 (%)**	**ACC (%)**	**AUC**	**F1 (%)**	**ACC (%)**	**AUC**	**F1 (%)**	**ACC (%)**	**AUC**
Chb01	18.10	34.77	0.32	29.28	39.44	0.38	17.97	35.58	0.32
Chb02	73.47	64.86	0.79	68.00	56.76	0.67	75.00	67.57	0.80
Chb03	54.17	52.79	0.53	57.48	59.37	0.60	40.62	50.64	0.51
Chb04	55.79	61.44	0.62	71.15	67.98	0.69	44.01	52.64	0.53
Chb05	47.65	63.07	0.70	60.14	69.51	0.75	70.69	73.34	0.74
Chb06	59.64	54.38	0.55	56.89	54.56	0.55	56.02	54.77	0.55
Chb07	67.77	56.88	0.63	67.33	56.29	0.61	67.99	58.00	0.63
Chb08	66.67	52.69	0.61	67.16	52.69	0.65	74.34	68.82	0.74
Chb09	59.19	63.51	0.64	68.13	63.79	0.65	74.88	70.21	0.73
Chb10	55.31	52.18	0.52	63.08	57.48	0.58	68.88	58.59	0.65
Chb11	97.62	97.70	0.98	95.12	95.40	0.96	96.47	96.55	0.96
Chb13	98.83	98.85	0.99	94.89	95.15	0.95	99.31	99.3	0.99
Chb14	13.92	53.74	0.76	45.17	64.59	0.80	31.86	59.48	0.78
Chb15	72.13	78.11	0.84	70.00	76.82	0.83	45.33	64.81	0.79
Chb16	70.81	77.37	0.84	76.09	80.73	0.86	95.89	95.77	0.96
Chb17	63.20	73.06	0.82	46.08	64.71	0.78	82.07	84.77	0.88
Chb18	68.87	76.13	0.83	70.14	77.02	0.84	76.29	80.85	0.86
Chb19	82.19	85.06	0.88	75.36	80.46	0.86	75.36	80.46	0.86
Chb20	39.71	62.13	0.77	12.60	52.83	0.67	9.48	52.38	0.71
Chb21	88.69	89.84	0.91	86.72	88.28	0.90	90.60	91.41	0.93
Chb22	90.21	91.09	0.92	90.52	91.35	0.93	93.73	94.10	0.95
Chb23	75.45	69.25	0.76	72.68	66.55	0.71	75.03	69.84	0.74
Average	64.52 ± 21.92	68.59 ± 16.72	0.73 ± 0.16	65.64 ± 19.81	68.72 ± 15.21	0.74 ± 0.14	66.45 ± 25.27	70.90 ± 17.63	0.75 ± 0.17

**Figure 9:**
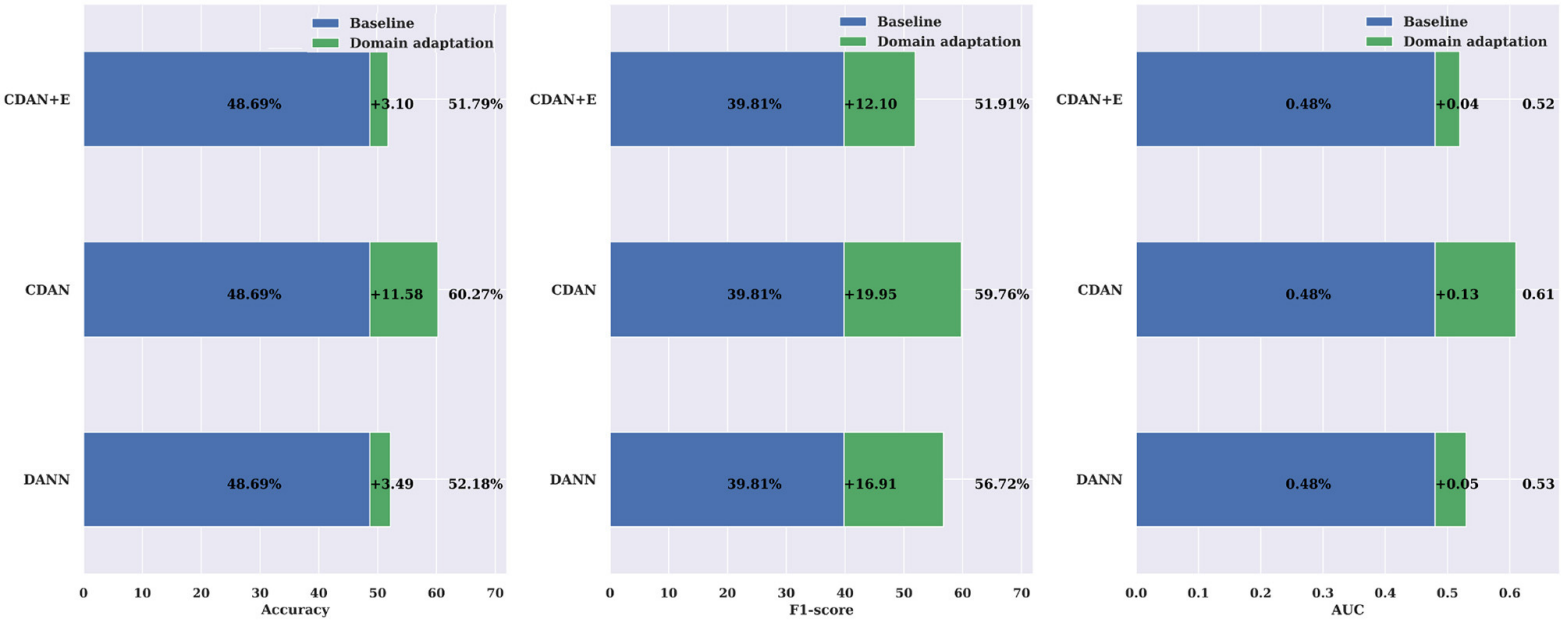
Domain adaptation for cross-subject modeling: a comparison between DANN, CDAN, and CDAN+E adaptation neural networks against the baseline cross-subject modeling using the leave-one-patient-out strategy on the SIENA dataset. Noticeable improvements over the baseline are consistently significant in terms of accuracy, F1-score, and AUC.

**Figure 10:**
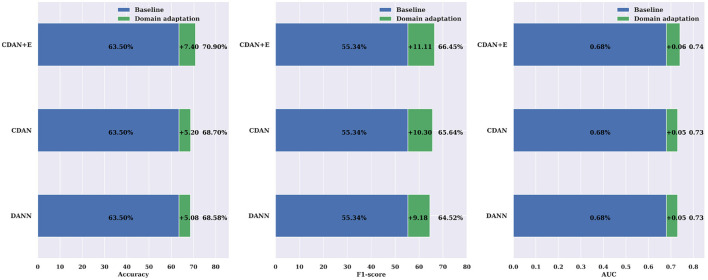
Domain adaptation for cross-subject modeling: a comparison between DANN, CDAN, and CDAN+E adaptation neural networks against the baseline cross-subject modeling using the leave-one-patient-out strategy on the CHB-MIT dataset. Noticeable improvements over the baseline are consistently significant in terms of accuracy, F1-score, and AUC.

The comparisons shown in Figure 9 reveal the importance of incorporating domain adaptation in cross-subject modeling compared to a traditional leave-one-patient-out approach. The CDAN method was found to significantly enhance the accuracy, F1-score, and AUC by 11.58%, 19.59%, and 0.13, respectively. The results were even better when evaluated on the CHB-MIT dataset (Figure 10), with an average improvement in accuracy, F1-score, and AUC of +7.40%, +11.11%, and +0.06%, respectively, using the CDAN+E method. Additionally, it was noted that the model's performance improved as the number of patients in the dataset increased.

A pertinent issue to consider is data under sampling to obtain a balanced dataset for improved learned model and reduced computational burden. Our examination of the SIENA dataset, with nearly balanced samples, demonstrated minimal impact from under-sampling. However, with the larger CHB-MIT dataset, potential implications become more evident. A key concern is the reduction of the majority class size, potentially impeding the model ability to capture diverse non-seizure patterns. This downsized dataset may result in decreased model performance, heightened sensitivity to noise, and biased representations of the majority class. Furthermore, under-sampling presents challenges in generalizing to new data and accurately identifying minority class instances like seizure events. A comparative analysis of results using the SIENA dataset, with limited data under sampling, and the extensively under sampled CHB-MIT dataset, suggests that under sampling does not significantly impact model performance or induce severe negative effects. Further investigations would be worthy of research, such as data augmentation, or neural networks of more representative objective functions. This line of investigation is crucial for ensuring the reliability and robustness of seizure prediction models in a scientific context.

Although accounting for patient sensitivity and specificity in model generalization ability is a problem worthy of consideration and research, it was not intended in the scope of this study. We acknowledge this as a limitation of the current study and mention it as a vein for future research that may significantly improve the method. Moreover, this study is subject to limitations inherent in the CHB-MIT and SIENA datasets, notably concerning gender, age, and ethnicity disparities. The datasets may not be representative of diverse populations, potentially introducing biases that affect the external validity of the findings. Gender imbalances may impact the generalizability of results, as conditions or responses to interventions may vary between genders. Additionally, uneven age distribution within the datasets may restrict the study's applicability to specific age groups, and ethnicity disparities may limit the generalizability of conclusions across different ethnic backgrounds. These inherent biases should be considered when interpreting the study's outcomes, emphasizing the need for cautious extrapolation to broader and more diverse populations.

In conclusion, the results of predicting seizures using cross-subject models show promising outcomes. Implementing seizure prediction models in clinical settings holds promise for early intervention and personalized treatment strategies. However, challenges include the necessity for high-quality, unbiased training data, and ensuring interpretability for healthcare professionals. Seamless integration into existing clinical workflows, ethical considerations regarding patient privacy, and legal compliance are crucial. The model's ability to generalize across diverse patient populations and adapt to external factors needs careful consideration. Navigating these challenges is essential to harness the potential of seizure prediction models while ensuring ethical and practical implementation.

## 5 Conclusion

In this study, our goal was to assess the generalization capability of a seizure prediction model to new patients. Therefore, we used a deep-learning architecture previously developed for patient-specific modeling in both multiple-subject and cross-subject scenarios. Our deep-learning architecture for multiple-subject seizure prediction was compared to existing state-of-the-art models and demonstrated superior performance. Despite the impressive accuracy of the model, its ability to generalize to new patients not in the dataset was uncertain. Thus, to better assess the model's performance and its ability to generalize to new patients, we employed a cross-subject modeling approach. This resulted in a noticeable decrease in performance when tested on open-access data. To overcome this issue, we investigated various domain adaptation methods to enhance the performance of cross-subject modeling. The results showed that all three methods (DANN, CDAN, and CDAN+E) significantly improved performance on both SIENA and CHB-MIT datasets.

Although this study realized significant progress in epileptic seizure prediction, there are currently limitations that could be addressed in future research. One limitation is the use of data under-sampling to deal with data imbalance, which not only impacts the computational cost of the training method but could also remove potentially valuable data relevant to seizure prediction. Another limitation is related to the size of the datasets used in the study. While the current amount of data allowed for a fair investigation of the problem, incorporating more data from additional EEG databases would offer further support to the findings and enhance the overall validity of the study. Moreover, it's crucial to acknowledge that the study refrained from using extensive statistical analyses, presenting an avenue for future investigations to delve deeper into the quantitative aspects of the predictive model.

In future research, we aim to address this limitations. This involves exploring alternative methods, beyond under-sampling, to balance our dataset. Techniques like over-sampling, employing synthetic data, or leveraging advanced neural networks designed for imbalanced datasets will be considered. Additionally, we intend to combine patient data from various datasets to address the challenge of limited data. This expanded dataset will be evaluated to better understand how well the model performs in different datasets.

## Data availability statement

Publicly available datasets were analyzed in this study. This data can be found here: The CHB-MIT Scalp EEG Database is available at https://physionet.org/content/chbmit/1.0.0/. The SIENNA Database is available at https://physionet.org/content/siena-scalp-eeg/1.0.0/.

## Ethics statement

The studies involving humans were approved by the dataset are publicly available. The studies were conducted in accordance with the local legislation and institutional requirements. Written informed consent for participation was not required from the participants or the participants' legal guardians/next of kin in accordance with the national legislation and institutional requirements.

## Author contributions

IJ: Conceptualization, Data curation, Formal analysis, Investigation, Methodology, Software, Supervision, Visualization, Writing – original draft, Writing – review & editing. LA-A: Investigation, Validation, Writing – review & editing. KH: Validation, Writing – review & editing. AM: Project administration, Resources, Validation, Writing – review & editing. NM: Funding acquisition, Resources, Validation, Writing – review & editing.
